# A Randomized Controlled Double Blind Trial of Ciclosporin versus Prednisolone in the Management of Leprosy Patients with New Type 1 Reaction, in Ethiopia

**DOI:** 10.1371/journal.pntd.0004502

**Published:** 2016-04-05

**Authors:** Saba M. Lambert, Digafe T. Alembo, Shimelis D. Nigusse, Lawrence K. Yamuah, Stephen L. Walker, Diana N. J. Lockwood

**Affiliations:** 1 Department of Clinical Research, Faculty of Infectious and Tropical Diseases, London School of Hygiene and Tropical Medicine, London, United Kingdom; 2 All Africa Leprosy Rehabilitation and Training (ALERT) Centre, Addis Ababa, Ethiopia; 3 Data Management, Armauer Hansen Research Institute (AHRI), Addis Ababa, Ethiopia; Fondation Raoul Follereau, FRANCE

## Abstract

**Background:**

Leprosy Type 1 (T1R) reactions are immune-mediated events leading to nerve damage and preventable disability affecting hands, feet and eyes. Type 1 Reactions are treated with oral corticosteroids. There is little evidence on alternative treatments for patients who do not respond to steroids or experience steroid adverse effects. We report the results of a randomized controlled trial testing the efficacy and adverse effect profile of ciclosporin and prednisolone (CnP) in comparison to prednisolone only (P) in patients with new T1R in Ethiopia. Ciclosporin is a potent immunosuppressant. Outcomes were measured using a clinical severity score, recurrence rate, adverse events and quality of life.

**Results:**

Seventy three patients with new T1R were randomized to receive CnP or P for 20 weeks. Recovery rates in skin signs was similar in both groups (91% vs 88%). Improvements in nerve function both, new and old, sensory (66% vs 49%) and motor (75% vs 74%) loss were higher (but not significantly so) in the patients on CnP. Recurrences rates of T1R (85%) were high in both groups, and recurrences occurred significantly earlier (8 weeks) in patients CnP, who needed 10% more additional prednisolone. Serious major and minor adverse events rates were similar in patients in the two treatment arms of the study. Both groups had a significant improvement in their quality of life after the study, measured by the SF-36.

**Conclusions:**

This is the first double-blind RCT assessing ciclosporin, in the management of T1R in Africa. Ciclosporin could be a safe alternative second-line drug for patients with T1R who are not improving with prednisolone or are experiencing adverse events related to prednisolone. This study illustrates the difficulty in switching off leprosy inflammation. Better treatment agents for leprosy patients with reactions and nerve damage are needed.

## Author Summary

Leprosy infection is cured with multi-drug therapy (MDT), but patients may develop immune mediated skin and nerve lesions. These immunological reactions lead to disability and deformity secondary to neuropathy. Prednisolone is the main drug used to treat reactions but is only partially effective and patients have a high rate of side effects. Identifying better agents for treating leprosy reactions is an important clinical goal. We tested the safety and efficacy of ciclosporin, an immunosuppressant used in many inflammatory conditions, in Type 1 reactions (T1R) in leprosy patients in Ethiopia. A double-blind randomized controlled clinical trial comparing the efficacy and adverse event profiles of ciclosporin and prednisolone was conducted in patients presenting with acute T1R. Patients on ciclosporin and prednisolone had similar improvements in clinical outcomes which were measured as skin and nerve function improvement. Both groups had a high rate of T1R recurrence (85%) and the patients on ciclosporin required more additional prednisolone to treat recurrences. We assessed patient quality of life and this was significantly improved with both treatments. This is the first assessment of patient quality of life in a leprosy patients trial. Ciclosporin may be a useful alternative in the treatment of T1R, but that the need for additional steroids decreases its value.

## Introduction

Leprosy is a chronic granulomatous infection principally affecting the skin and peripheral nerves caused by the obligate intracellular organism *Mycobacterium Leprae* [1]. In 2014, the WHO reported 213 899 new cases globally [2]. Multi-drug therapy (MDT) cures the infection by *Mycobacterium leprae*. Although the bacteria may be eliminated, the damage done to nerves by the bacteria and by consequent immunological reactions leads to very visible and stigmatizing disabilities and deformities.

Type 1 reactions (T1R) affect up to 30% of patients with borderline leprosy [3]. Although T1Rs can occur at any time, the frequency is higher in the first six months of MDT treatment [4]. T1R manifest clinically with erythema and oedema of skin lesions and tender peripheral nerves with loss of nerve function. Skin lesions become acutely inflamed and oedematous. Inflammation is usually in pre-existing lesions, but not all the lesions may be involved. Oedema of the hands, feet and face can also be a feature of a reaction but systemic symptoms are unusual. Nerves can become swollen, painful and tender. Acute neuritis may also occur without evidence of skin inflammation. The inflammatory process in leprosy reactions leads to nerve function impairment (NFI) which if not treated rapidly leads to permanent loss of nerve function causing peripheral sensory and motor neuropathy. Recurrent T1Rs can lead to further nerve damage [5]. Progressive NFI can also occur in the absence of a reactional state, so the history of timing of symptoms aids to differentiate from NFI due to a reaction.

T1Rs are the result of spontaneous enhancement of cellular immunity and delayed hypersensitivity reactions to *M*.*leprae* antigens presented by macrophages and dendritic cells in the skin and by Schwann cells on nerves [6,7]. Immuno-suppression is required to control the symptoms and signs of T1R, but this management remains challenging. Oral prednisolone, the drug of choice, has frequent side effects and approximately 40% of individuals with T1R do not show clinical improvement [8,9]. There is a lack of evidence for efficacious and safe second line treatments for T1R.

Ciclosporin is a potent immuno-suppressant that has been widely and successfully used as a treatment for psoriasis, Behcet’s disease, rheumatoid arthritis, inflammatory bowel disease and transplantation. Given that ciclosporin selectively inhibits the activation of CD4 T cells and the expression of cytokines such as IL-2 and TNF-α [10], it was thought to be useful in the treatment of T1R. Three case studies have been published [11,12], showing good response to ciclosporin and delayed recurrence of T1R. An uncontrolled pilot study was carried out assessing the efficacy of ciclosporin in severe T1R in Ethiopian and Nepali patients [13]. In the Ethiopian part of the study, performed in ALERT Hospital, Addis Ababa, ciclosporin was given to 33 patients with T1R for three months in a dose range of 5–7.5mg/kg/day. This led to improvements in skin lesions in 85% of patients and 45% of patients had improvement in nerve pain and tenderness. Sensory nerve impairment improved in 45% of Ethiopian patients and motor function impairment in 53% of patients. Almost 88% of Ethiopian patients needed the higher dose of ciclosporin to show improvement partly because of the severity of the reaction. The study showed that in those patients treated with high-dose ciclosporin, 53% of patients with sensory impairment and 60% with motor impairment improved. A few Ethiopian patients with NFI of greater than 6 months duration, responded to ciclosporin. This was an encouraging result as in many leprosy endemic countries patients present late with chronic NFI. Almost 70% percent of Ethiopian patients developed new signs of reaction after stopping treatment, suggesting that they would benefit from a treatment period longer than three months. In the Nepali study, ten patients treated with ciclosporin were compared to a similar group of patients treated with prednisolone. Improvement in skin lesion was at 87.5% in the ciclosporin group compared to 74% in the prednisolone group. Similarly the ciclosporin group showed 83% improvement in sensory testing compared to 22% in the prednisolone group.

Few ciclosporin side effects were seen in the two clinical trials conducted in T1R. Of the 33 Ethiopian patients, three developed hypertension; of the ten Nepali patients one developed jaundice (possibly dapsone related), two developed raised serum creatinine levels and two other patients developed mild side effects (loss of appetite and indigestion controlled with antacids).

The results of the above studies were encouraging as it appeared that ciclosporin monotherapy may be an effective alternative treatment in prednisolone-resistant or prednisolone-dependent cases of T1R. The study recommended using higher doses of ciclosporin (7.5mg/kg/day) in future studies, longer periods of treatment, as well as tapering the drug slowly or adding low dose prednisolone to prevent relapse.

We tested our hypothesis that ciclosporin would be as effective as prednisolone in the treatment of patients with leprosy reactions and nerve function impairment and that patients treated with ciclosporin would have fewer side effects than patients treated with prednisolone. A randomized controlled trial comparing ciclosporin and prednisolone in the treatment of leprosy T1R was designed and conducted.

## Methods

### Study design and participants

A double-blind controlled trial was conducted randomizing patients with new and recent onset T1R to treatment with either ciclosporin and prednisolone or prednisolone alone. Patients were recruited at the ALERT hospital leprosy clinic, in Addis Ababa, Ethiopia.

### Case definitions

**Type 1 Reaction** (T1R) was diagnosed when a patient with leprosy had erythema and oedema in skin lesions and/or neuritis. A patient could have skin reaction only, a nerve reaction only or a skin and nerve reaction.

**Neuritis** was diagnosed when a leprosy patient had any of the following on history or examination:

- Spontaneous nerve pain, paraesthesia or nerve tenderness
- New sensory or motor impairment of recent onset
- Mixed sensory and/or motor impairment with nerve tenderness.

**Nerve function impairment** (NFI) was defined as clinically detectable impairment of sensory or motor nerve function using the definitions below [14].

**New NFI** was defined as less than six months duration of reduction in sensory or motor function on history or examination.

**Motor loss** was defined by a decrease in voluntary muscle testing (VMT) score, by 1 point or more from the normal score of 5, using the modified MRC scale.

**Sensory loss** was defined by a decrease in sensation as measured by Semmes Weinstein monofilament testing. In the hands, this was defined as not being able to perceive the 0.2gm monofilament at 2 points out of 3 in each nerve of the hand. In the feet, this was defined as not being able to perceive the 2gm monofilament at 3 out of 4 sites of the foot.

**Silent neuropathy** (SN): A patient had silent neuropathy when he/she had sensory and/or motor impairment of recent onset (less than six months duration) in an area innervated by one or more nerve without signs of a reaction (RR or ENL) or nerve pain with or without tenderness.

**T1R recurrence or flare-up** was defined as an increase in skin severity score to 4 or more out of 9 AND/OR an increase in NFI defined as worsening of VMT by one point in two or more muscles, or by 2 points in one muscle and/or worsening of ST: decreased sensation in at least two out of 3 points per nerve on the hand and/or 3 or more points on the feet. NB: nerve tenderness was not part of the definition for T1R recurrence.

**NFI outcomes** were defined clinically as (based on Marlowe study [13]:

1. **Recovered** when the motor or sensory function returned to normal;
2. **Improved** when the motor function improved by the VMT improving by one point in two or more muscles or by 2 points in one muscle and /or the sensory function improved by at least two out of 3 points per nerve on the hand and/or 3 or more points on the feet;
3. **Not improved** when no changes where recorded in either VMT or ST;
4. **Worse** when the motor function or sensory function where found to be decreased by any point on VMT and/or ST;
5. **Remained stable after treatment** when the final assessment at week 28 or 32 showed that motor and or sensory function was similar or better compared to the end of treatment assessment at week 20;
6. **Relapsed after treatment** when the final assessment at week 28 or 32 showed that motor and or sensory function was worse compared to the end of treatment assessment at week 20.

**Clinical Severity Score**: used to assess T1R severity (21 items; range of 0–63). The maximum score possible for skin (A), sensation (B) and motor function (C) are 9, 24 and 30 respectively. Mild T1R is characterised by a score of 4 or less; moderate T1R by a score between 4.5 and 8.5 and severe T1R is a score of 9 or more. This Severity Scale for T1R based, on the INFIR clinical severity scoring system, was developed and prospectively validated in Bangladesh and Brazil [15]. It has so far been used in clinical trials on intravenous methylprednisolone [16], on azathioprine [17] and in the on-going TENLEP studies [18].

### Eligibility

Participants (aged between 18 and 65 years and weighing more than 30 kg) were recruited from the leprosy clinic in ALERT Hospital, Addis Ababa, Ethiopia. Individuals with newly diagnosed T1R or neuritis were eligible for entry in the trial.

### Exclusion criteria

The following individuals were excluded: those unwilling to give consent or return for follow up; those with severe active infections such as tuberculosis or severe inter-current disease; HIV positive individuals; pregnant or breastfeeding women. Women of reproductive age not willing to use contraception for the duration of the study were also excluded.

### Treatment arms

The participants were randomly allocated to receive the standard ALERT hospital prednisolone regimen for T1R or the ciclosporin (Cn) arm (Table 1). We theorized that given the slow onset of action of ciclosporin compared to prednisolone and high relapse rate of T1R, the most effective regimen in leprosy reaction would be an initial ciclosporin dose of 7.5mg/kg/day, divided in two doses, gradually tapered down over a total period of 20 weeks and adding prednisolone cover for the first four weeks of treatment. The TIR patients on the prednisolone arm (P) would get 20 weeks of a gradually reducing course of prednisolone only.

**Table 1:** Treatment regimen for T1R trial.

	Prednisolone alone arm	Ciclosporin and Prednisolone arm
**Week 1**	Prednisolone 40mg+ PC*	Ciclosporin 7.5mg/kg + Prednisolone 40mg
**Week 2**	Prednisolone 40mg + PC	Ciclosporin 7.5mg/kg + Prednisolone 40mg
**Week 3**	Prednisolone 35mg + PC	Ciclosporin 7.5mg/kg + Prednisolone 20mg
**Week 4**	Prednisolone 35mg + PC	Ciclosporin 7.5mg/kg + Prednisolone 10mg
**Wk 5 & 6**	Prednisolone 30mg + PC	Ciclosporin 7.5mg/kg + PP**
**Wk 7 & 8**	Prednisolone 25mg + PC	Ciclosporin 7.5mg/kg + PP
**Wk 9–12**	Prednisolone 20mg + PC	Ciclosporin 7.5mg/kg + PP
**Wk13–16**	Prednisolone 15mg + PC	Ciclosporin 6mg/kg + PP
**Wk17–18**	Prednisolone 10mg + PC	Ciclosporin 4mg/kg + PP
**Wk19–20**	Prednisolone 5mg + PC	Ciclosporin 2mg/kg + PP
**Total prednisolone**	3080mg	770mg

*PC = placebo ciclosporin

**PP = placebo prednisolone

Weight adjusted medication cards for each treatment arm were designed for the pharmacist, using a 10 kilogram range in patient weight. A double placebo system was used because of the different formulation of prednisolone (pink tablets) and ciclosporin (brown capsules). Each placebo was identical to its active counterpart and each participant took a combination of brown capsules and pink tablets as an essential way of blinding both patients and study physicians. Prednisolone tablets and prednisolone placebo (PP) tablets were produced by Ethiopian Pharmaceuticals Manufacturing Factory (EPHARM), in Addis Ababa, Ethiopia. Both were analysed for active ingredient by Dr Harparkash Kaur at LSHTM. Ciclosporin capsules (Panimune Bioral) and ciclosporin placebo (PC) capsules were produced by Panacea-Biotec Ltd, Solan, India and were provided with a certificate of analysis.

### Clinical assessments

A full history was taken and clinical examination performed. Nerve function was assessed at each visit, by one of three trained physiotherapists. Sensory testing was performed with five Semmes-Weinstein monofilaments at designated test sites on hands and feet. Voluntary muscle power was graded using the modified Medical Research Council scale. The results of the examination findings were recorded and a Clinical Severity Score calculated using the severity scale. Severity of reaction was also recorded as mild, moderate or severe by a second physician’s opinion blinded to the Clinical Severity Score.

Laboratory investigations consisted of the following: slit skin smears for bacterial index, full blood count, HIV test, renal function, liver function tests, glucose, erythrocyte sedimentation rate (ESR), urinalysis and a stool specimen examined for ova, cysts and parasites. A skin biopsy was performed for Ridley-Jopling classification. Symptomatic screening for TB was carried out by chest x-ray and sputum samples for acid fast bacilli as necessary.

All individuals received three days of albendazole 400mg daily to reduce the risk of hyper-infection with *Strongyloides stercoralis* at enrolment.

Women of reproductive age were tested for pregnancy and contraception was prescribed, usually the oral contraceptive pill and condoms.

Assessments were carried out at weeks 2, 4, 6, 8, 12, 16, 20, 24, 28, and 32 from enrolment. Assessment consisted of focussed questions about specific symptoms and adverse effects. The clinical examination including weight and blood pressure was repeated. Blood tests (full blood count, renal function and liver function), and urinalysis were carried out at each visit.

Quality of life was assessed with a validated Amharic translation of the SF-36 health-related quality of life assessment tool [19] at recruitment and at week 28. Each patient’s quality of life is graded with two scores: a physical score (PCS) and a mental score (MCS), which in turn are composed of four subscales each.

### Outcome measures

The primary outcome measure was the change in Clinical Severity Score and in clinical nerve function impairment and at week 4, 20, and 28 for patients in each treatment arm. Secondary outcomes were:

1. Mean time to recurrence of T1R for patients in each treatment arm
2. Number of T1R recurrence episodes per patient in each treatment arm:
  - Whilst on treatment (week 1–20)
  - During follow-up (week 21–32)
3. Severity of T1R recurrence for patients in each treatment arm:
  - Whilst on treatment (week 1–20)
  - During follow-up (week 21–32)
4. Amount of extra prednisolone for patients in each treatment arm:
  - Whilst on treatment (week 1–20)
  - During follow-up (week 21–32)
  - Total
5. Frequency of adverse events in patients in each treatment arm
6. Difference in score in Quality of Life assessment between start and end of treatment for patients in each treatment arm

Criteria for using additional prednisolone were defined as sustained deterioration in nerve function nerve pain unresponsive to analgesics for a period of at least two weeks; new erythematous and raised skin patches; deterioration in nerve function which the study doctors believe requires immediate additional prednisolone and ENL flare-up with the appearance of new subcutaneous nodules.

As the study was double-blinded, regimen for additional prednisolone depended on the time at which the reaction flare-up occurred. If the reaction recurrence was within the first ten weeks of treatment or there was facial involvement, extra prednisolone was added to make up a total of 40mg (with the pharmacist deciding on the exact additional dose of prednisolone required) and then tapered according to the original regimen. If T1R recurrence was after the first ten weeks of treatment, then prednisolone 20mg was added and tapered down according to the original regimen. The physician could prescribe more additional prednisolone if the reaction was severe.

Adverse events were enquired about at each visit using a standardized form with anticipated adverse events attributable to prednisolone and ciclosporin. Any other adverse events reported by the participant or identified by the physicians were also recorded. Major adverse events were defined as any event leading to admission or prolonged admission, study un-blinding or death. Amongst these were included psychosis, severe infection including tuberculosis, peptic ulcer, glaucoma, cataract, diabetes mellitus, severe hypertension and haematological abnormalities. Minor adverse events were defined as moon face, acne, hirsutism, gum hyperplasia, fungal infections, gastric pain requiring antacids or any other minor adverse event not requiring admission to hospital or un-blinding. Patients who experienced blurred vision were referred to the ophthalmologist for ophthalmic review and had their serum glucose checked. Three study physicians (blinded to each other’s decision) reviewed each adverse event and decided whether it was linked to prednisolone or ciclosporin. Adverse events were also graded by severity, using the Common Terminology Criteria for Adverse Events [20] grading system.

### Randomisation and masking

Eligible individuals were recruited consecutively and randomly assigned in 1:1 ratio (block size of four), with a computer-generated randomisation list, to one of the two treatment arms. A standard envelope system was used for allocation concealment. The envelopes were prepared by an individual who had no other involvement in the study. The allocation procedure was done by the pharmacist who had no clinic contact and was the only individual aware of the treatment allocation. All study participants, physicians, nurses, ward staff, laboratory staff and the physiotherapists were blinded to the allocation. The allocation code was revealed to the researchers once the study was completed, except in the case of a serious adverse event necessitating un-blinding.

### Statistical analysis

The sample size, based on the Hypothesis of Non-Inferiority, was calculated with the study statistician, in consultation with ALERT hospital physicians. Prednisolone is known to show an improvement of 60% in nerve function in new T1R. Given that the true mean cure rates of the treatment agents and the active control are θ1 = θ2 = 60%, the non-inferiority margin was selected to be δ = 0.25. The sample size was calculated using a power of β = 80% and significance of α = 0.05, giving us a sample of n = 48 in each arm respectively.

The data was entered in Access database and analysed using the Statistical Package for the Social Sciences (SPSS version 20. SPSS Inc., Chicago, Illinois). An intention to treat analysis (ITT) was used for calculating the effects of treatment on individuals in each group and t tests and ANOVA (analysis of variance) were used as appropriate. The Mann-Whitney U test was used for all statistical tests of continuous variables and Fisher’s exact test was used to compare dichotomous variables.

### Ethics statement

The studies were performed according to the Helsinki Declaration (2008 revision) and approved by the Ethics Committee of the London School of Hygiene and Tropical Medicine (5376), the ALERT and AHRI Ethical Review Committee (AA/ht/248/09), the National Ethics Review Committee of Ethiopia (RDHE/34-90/2009), and the Drug Administration and Control Authority of Ethiopia (02/12/70/926). All staff involved underwent Good Clinical Practice training and an independent Data and Safety Monitoring Board reviewed the study design and the safety and efficacy data. The study is registered with ClinicalTrials.gov: NCT00919815.

Written informed consent was obtained in Amharic or if the patient spoke a different Ethiopian language, then the information and consent forms were translated verbally into the appropriate language before signing the consent form.

## Results

**Fig 1:**
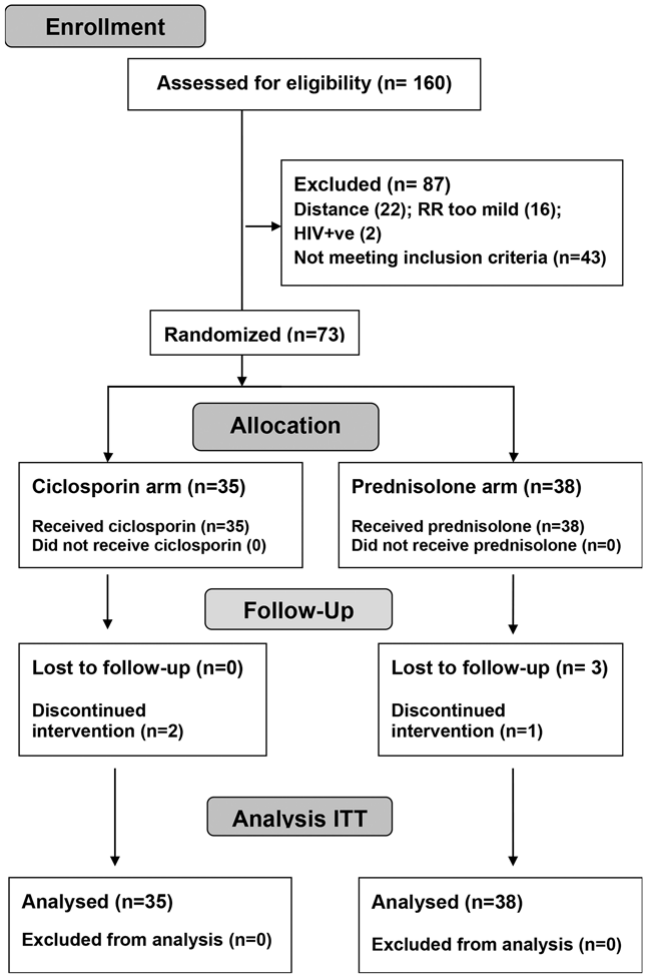
CONSORT flow diagram.

Seventy three patients with recent onset T1R were enrolled into the trial between 12^th^ August 2011 and 25^th^ December 2012. The final assessment was completed on 24^th^ July 2013. Thirty five individuals were randomized to the ciclosporin arm, and 38 to the prednisolone arm. The participant flow is shown in the CONSORT flow diagram (Fig 1). The trial was terminated before full recruitment numbers were reached because of slower recruitment rates than anticipated.

Six patients did not complete the intervention medication. Three patients in the prednisolone arm did not attend for review at week 2 or 4. One other patient in the prednisolone arm had a serious adverse event which led to un-blinding at week 6. He was removed from the study at his request and continued to take prednisolone at a different facility. Two patients in the ciclosporin arm were discontinued from the study, one for non-adherence at week 12, and the second patient had a serious adverse event on week 6 which necessitated un-blinding and discontinuation of ciclosporin. He continued the study on prednisolone.

The two groups of patients with T1R were not significantly different with respect to sex, age, Ridley-Jopling classification, or treatment with MDT (Table 2).

**Table 2:** Description of study participants in each arm.

Participants with new T1R		Ciclosporin (n = 35)	Prednisolone (n = 38)
**Sex**	**Women: men**	**7:28**	**8:30**
**Median age (years)**		**27**	**34**
**Median weight (kg)**		**52**	**54**
**Clinical Ridley- Jopling**	**TT**	**0**	**1**
**classification**	**BT**	**27**	**23**
	**BB**	**2**	**6**
	**BL**	**5**	**7**
	**LL**	**0**	**1**
	**PNL**	**1**	**0**
Mean BI*	**at diagnosis**	**0.7**	**0.9**
	**at recruitment**	**0.2**	**0.1**
**MDT status**	**Started at enrolment**	**25**	**22**
	**Current**	**4**	**7**
	**Completed**	**6**	**9**
**Co-morbidities**		**2 foot ulcer**	**1 Foot ulcer**
			**3 skin ulcer**
		**2 skin ulcers**	
		**2 fungal infections**	
		**2 conjunctivitis**	
		**1 intrahepatic cholecystiasis**	
**EHF score (mean)**		**3.94**	**3.84**

*Mean BI = group mean of each patient’s mean BI

PNL = pure neural leprosy

Of the 73 participants, 50 had BT leprosy (70% had a negative BI) and 12 patients had BL leprosy. Of all participants presenting with T1R, 64% were newly diagnosed with leprosy. In these patients the signs and symptoms of the reaction were the reason for seeking medical assistance.

### Reaction type

The two groups did not differ significantly in respect of reaction type, or mean number of enlarged and tender nerves per patient (Table 3). There was a significant difference in the duration of NFI between patients recruited to the two groups (Chi Square, p = 0.039). Twice as many patients in the ciclosporin arm reported isolated new NFI but in the prednisolone arm, there were more patients reporting combination of old and new NFI.

**Table 3:** Reaction type and nerve involvement in study participants.

Participants with new T1R		Ciclosporin	Prednisolone	P value
		(n = 35)	(n = 38)	
**Reaction type**	**Skin only**	**4**	**8**	**0.541**
	**Skin and nerves**	**28**	**27**	
	**Nerve only**	**3**	**3**	
**Facial patches**		**29**	**25**	**0.164**
**Peripheral Oedema**		**30**	**28**	**0.414**
**Reported NFI at baseline**	**None**	**3**	**9**	**0.039**
	**New**	**20**	**10**	
	**Old**	**4**	**4**	
	**Mixed old and new**	**8**	**15**	
**Mean number of enlarged nerves per patient**		**9**	**8.5**	**0.306**
**Mean number of tender nerves per patient**		**4.7**	**3.6**	**0.168**

Type 1 reaction occurring in both skin and nerves was present in 76% of participants, whilst 16% had reaction affecting skin only and 8% nerves only. 74% of patients had inflamed facial patches and 80% had peripheral oedema on examination.

### Duration and severity of T1R

Patients in the two treatment arms had similar duration of reported T1R symptoms prior to presenting at the clinic (p = 0.2). Severity of T1R, assessed both by specialist opinion and by the Clinical Severity Score, was not significantly different between the two groups (Table 4).

**Table 4:** Duration and severity of T1R in study participants.

Participants with new T1R		Ciclosporin	Prednisolone	P value
		(n = 35)	(n = 38)	
**Reported mean duration of T1R symptoms (days)**		**61.5 (6–180: median 58)**	**49.6 (5–150: median 44)**	**0.2**
**Severity by specialist opinion**	**Moderate**	**1**	**3**	**0.667**
	**Severe**	**34**	**35**	
**Severity by Clinical Severity Score (mean)**	**Score A (skin)**	**5.74**	**5.11**	**0.19**
	**Score B (sensation)**	**8.53**	**7.77**	**0.53**
	**Score C (motor)**	**9.37**	**6.92**	**0.58**
	**Total CSS score**	**22.96**	**19.79**	**0.36**

### Nerve involvement

The 73 patients recruited had a total of 876 peripheral nerves examined. Nerve function impairment of less than 6 months duration (new NFI) was reported for 308 nerves (35%). A further 24% of nerves were reported to have been impaired for longer than 6 months (old NFI). In both old and new NFI, sensory loss was more frequent than motor loss or mixed loss. Of the nerves examined, 72% were enlarged, and 34% of nerves were tender on palpation. A larger proportion of nerves were impaired in the ciclosporin group patients (68% vs. 52%) and this group had significantly higher proportion of purely sensory and mixed sensory/motor types of new NFI (p = 0.0387)

The ulnar nerves were found to be both the most frequently enlarged and tender nerves, followed by the lateral popliteal, radial cutaneous and posterior tibial nerves. Nerve tenderness was present in 300 nerves and was more common in the ciclosporin group (40% vs. 29%). Apart from a higher number of affected sensory nerves in the ciclosporin group, there was no major significant difference between the two groups of patients with newly diagnosed T1R, recruited to the study.

### Primary outcome: Change in Clinical Severity Score and in clinical nerve function impairment

**Fig 2:**
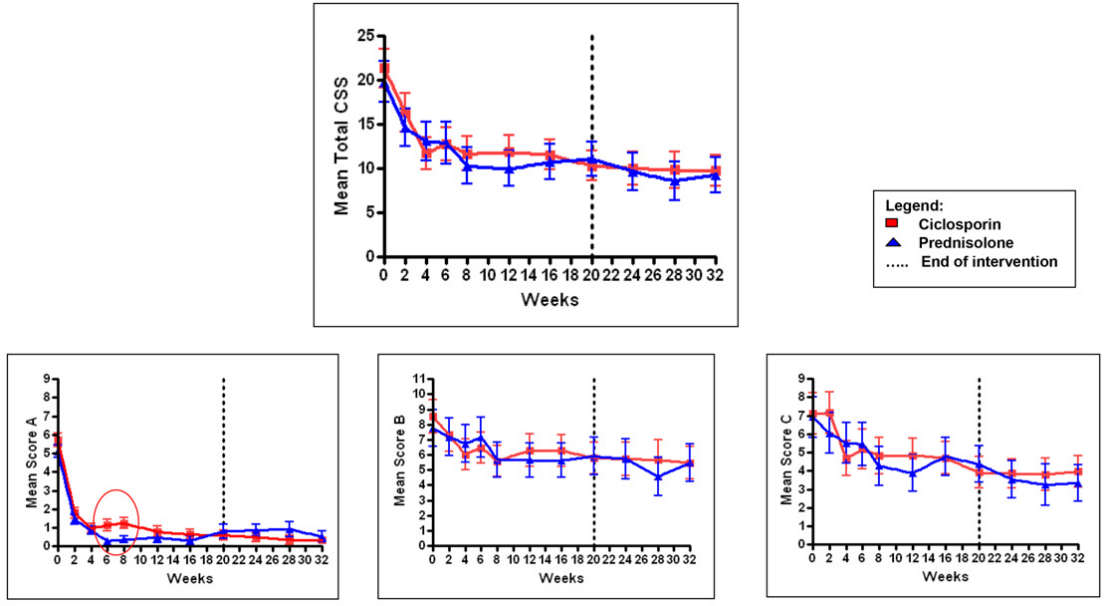
Group mean and standard error in Clinical Severity Scores with time by treatment arm. Score A (skin), Score B (sensation) and Score C (motor). (0 marks the area in Score A where the difference between the treatment arms is significant).

The change in group mean Clinical Severity Score over time for patients in each arm of the trial is shown in Fig 2. Changes in the three sub-scores are also shown. Variation in group mean T1R severity scores during the 32 weeks and between the two treatment arms, was assessed by ANOVA. Patients in both treatment arms had large and statistically significant improvement with time in all four scores (p<0.000). This is consistent with a good clinical response with both treatments.

There was no significant difference in all four severity scores between the two treatment arms over the 32 weeks (Score A, p = 0.241; Score B, p = 0.664, Score C, p = 0.749 and Clinical Severity Score, p = 0.531).

In the ANOVA week by week breakdown, patients on the ciclosporin arm showed significantly higher skin score (A), at weeks 6 and 8 (p<0.000). This was probably due to a greater number of patients in the ciclosporin arm experiencing a flare-up in skin reaction at this time.

**Fig 3:**
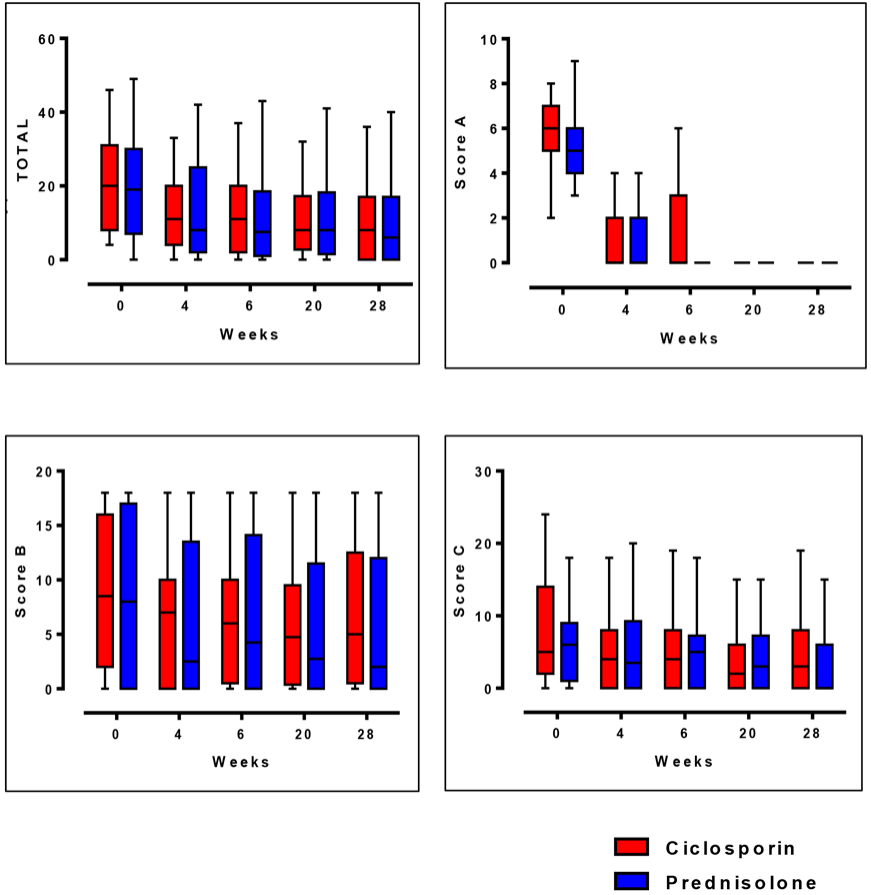
Median and inter-quartile ranges in Clinical Severity Scores.

The difference between the two treatment groups in median improvement of Clinical Severity Scores were compared at week 0, 4, 6, 20, and 28 as shown in Fig 3. These time periods were deemed important, as at week 4, the prednisolone in the ciclosporin arm is stopped; at week 6 is the steroid free period for those on the ciclosporin arm; at week 20 the intervention period ends, and week 28 represents the end of the study.

All four components of the severity scores show a downward trend, suggesting improvement in both groups of patients. The largest and sustained decrease in score occurs in the skin (A). At week 6, the difference in skin score between the two treatment arms is evident with the patients in the ciclosporin arm having a wider range in score despite a similar median score. Throughout the 32 weeks in the study, the median sensory score (B) does not reach the score of 0, which represents intact sensation.

Analysis by patient and by nerves were also done to assess the improvement in T1R in patients treated with either ciclosporin or prednisolone.

The general outcome for patients (Table 5) was decided by study physician assessment on review of patient notes and taking into account the changes in skin as well as nerves between week 0 and week 20, the end of the intervention period. There is no significant difference in all six clinical outcomes listed in Table 5 between the patients in the two treatment arms. Clinical outcomes in the follow-up period were recorded as those that maintained improvement and those that relapsed at the end of treatment. A larger proportion of patients appears to be maintaining improvement after the end of the intervention period in the ciclosporin arm (67% vs.39%, p = 0.044).

**Table 5:** Clinical outcome in patients with new T1R.

Clinical outcome in patients		Ciclosporin		Prednisolone			P value
Number of patients enrolled		35		38			
**General T1R status**							
** **	No (%) recovered	1	**3%**	4		**11%**	0.254
** **	No (%) improved	31	**89%**	26		**75%**	
** **	No (%) not improved	3	**8%**	5		**14%**	
** **	*No (%) maintained improvement after Rx*	*22*	***67%***	*12*		***39%***	*0*.*044*
** **	*No (%) relapsed after Rx*	*11*	***33%***	*19*		***61%***	
**Skin signs**							
** **	No (%) recovered	32	**91%**	31		**88%**	0.33
** **	No (%) improved	3	**9%**	2		**6%**	
** **	No (%) no change	0	**0**	2		**6%**	
** **	*No (%) maintained improvement after Rx*	*28*	***85%***	*21*		***68%***	*0*.*143*
** **	*No (%) relapsed after Rx*	*5*	***15%***	*10*		***32%***	
**Sensation**							
** **	No (%) recovered	1	**3%**	0	**0**		0.204
** **	No (%) improved	22	**63%**	17	**49%**		
** **	No (%) no change (normal)	5	**14%**	12	**34%**		
** **	No (%) not improved	7	**20%**	6	**17%**		
** **	*No (%) maintained improvement after Rx*	*26*	***79%***	*23*	***74%***		*0*.*771*
** **	*No (%) relapsed after Rx*	*7*	***21%***	*8*	***26%***		
**Motor function**							
** **	No (%) recovered	16	**46%**	14	**40%**		0.957
** **	No (%) improved	10	**29%**	12	**34%**		
** **	No (%) no change (normal)	6	**17%**	6	**17%**		
** **	No (%) not improved	3	**8%**	3	**8%**		
** **	*No (%) maintained improvement after Rx*	*27*	***82%***	*29*	***94%***		*0*.*259*
** **	*No (%) relapsed after Rx*	*6*	***18%***	*2*	***6%***		
**Nerve tenderness**							
** **	No (%) improved	25	**71%**	22	**63%**		0.285
** **	No (%) no change (normal)	7	**20%**	12	**34%**		
** **	No (%) not improved	3	**9%**	1	**3%**		
** **	*No (%) maintained improvement after Rx*	*28*	***85%***	*23*	***74%***		*0*.*359*
** **	*No (%) relapsed after Rx*	*5*	***15%***	*8*	***26%***		
**EHF Disability Score**							
** **	No (%) improved	23	**66%**	18	**51%**		0.168
** **	No (%) no change (normal)	9	**26%**	16	**46%**		
** **	No (%) worse	3	**8%**	1	**3%**		

T test done with Chi Square

**Fig 4:**
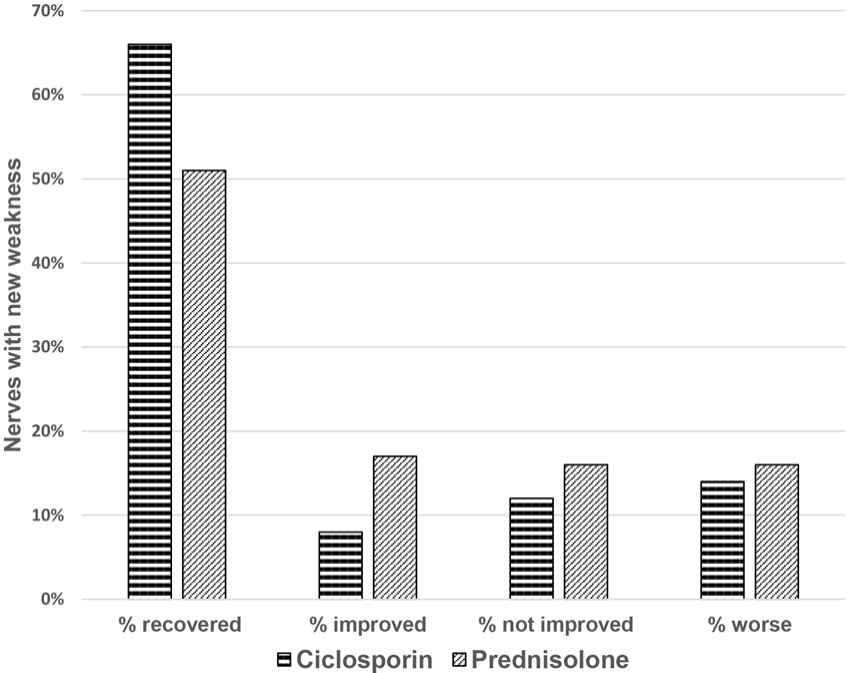
Motor function change in nerves with new weakness.

The change in motor function, between baseline and the end of intervention, in nerves with reported weakness of less than six months duration is not significantly different between the two study arms (p = 0.085). Fig 4 illustrates that motor function in both treatment arms recovered or improved in a large proportion of nerves (74% in the ciclosporin arm and 68% in the prednisolone arm (one tailed t test: p = 0.043).

**Fig 5:**
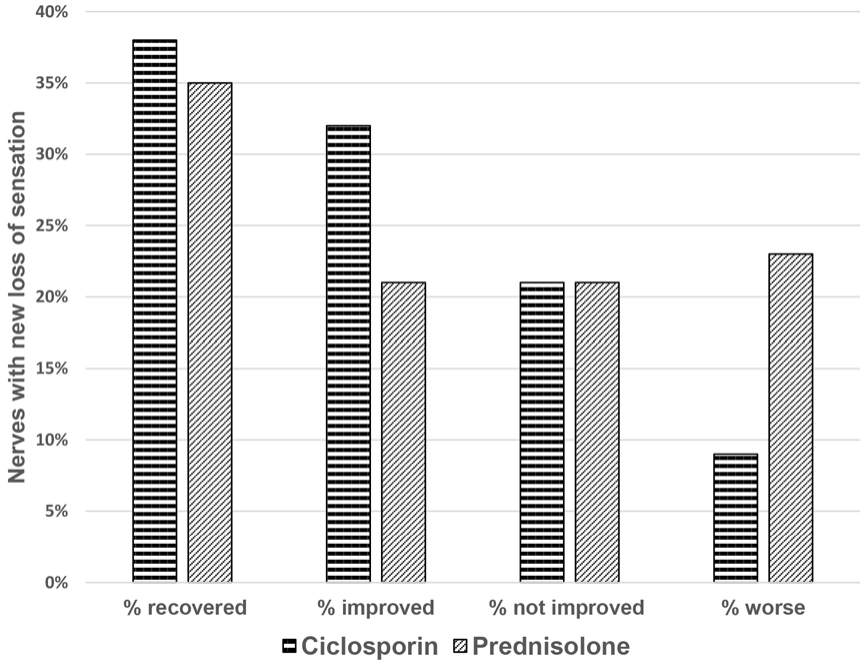
Sensory function change in nerves with new loss of sensation.

70% of nerves with sensory loss reported as being of less than six months duration in the ciclosporin arm and 56% in the prednisolone arm improved or recovered (Fig 5). Patients who received ciclosporin and prednisolone had better improvement in nerve function impairment than those who received prednisolone only (one tailed t test: p = 0.038).

Patients in both treatment arms had their nerves assessed three months after the end of the intervention and improvement in nerve function was maintained in the majority of patients. Motor function remained stable in 88% (Cn arm) and 76% (P arm), and sensory function in 78% (Cn arm) and 79% (P arm).

Nerves reported to have been impaired for longer than six months also showed improvement. Of the nerves with old motor function 37% in the Cn arm and 39% in the P arm recovered or improved. Of the nerves with old loss of sensation, 46% in the Cn arm and 36% in the P arm recovered or improved.

### Secondary outcomes

#### Mean time to recurrence of T1R

Fifty nine out of 69 (85%) patients recruited with new T1R had a T1R recurrence.

Of the 73 patients recruited to the study, the three who withdrew from the prednisolone arm early in the study, and one patient in the prednisolone arm who then had ENL recurrences only throughout the 32 weeks in the study have been removed from this analysis. Ten patients had no T1R recurrence during the 32 weeks in the study: five patients in the ciclosporin arm and five in the prednisolone arm. Six patients, two in the ciclosporin arm and four in the prednisolone arm, had an ENL episode, in the 32 weeks in the study. These patients experienced both ENL and T1R, and so have been retained in the analysis.

**Fig 6:**
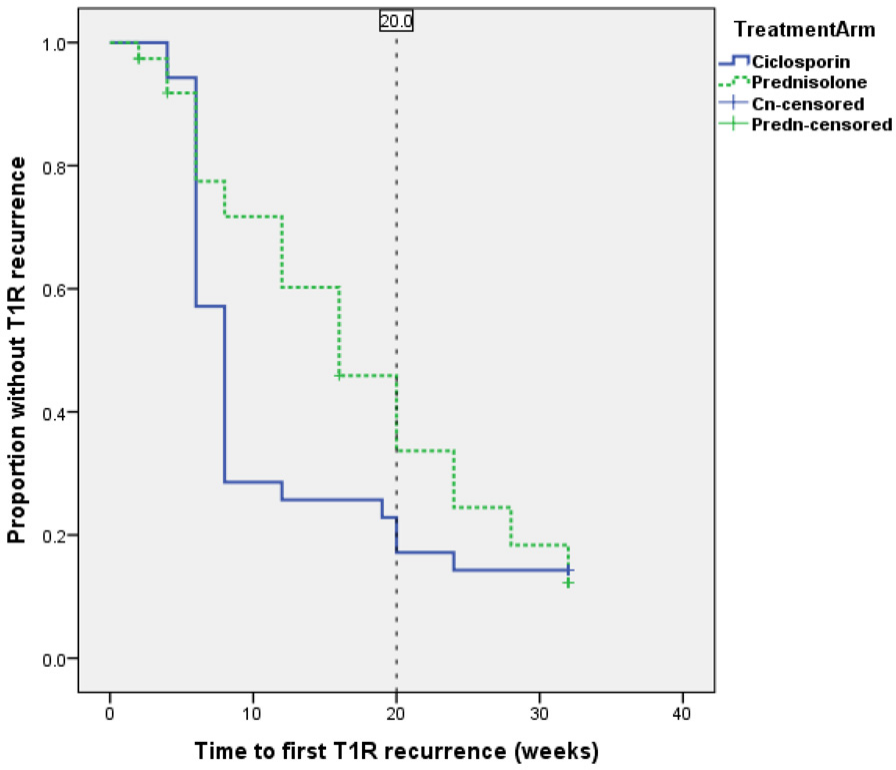
Survival curve for patients without a T1R recurrence.

The cumulative probability of T1R recurrence at a given point of time is shown on a Kaplan-Meier survival curve and there is no statistically significant difference between the two treatment arms (Log Rank- Mantel Cox, p = 0.157) (Fig 6).

The mean time to first episode of T1R recurrence was 8.7 weeks (median = 8) in the ciclosporin group and 15.2 (median = 16) weeks in the prednisolone group. The earlier time to first recurrence in the patients on the ciclosporin arm was statistically significant (Mann-Whitney U Test, p = 0.0058).

**Fig 7:**
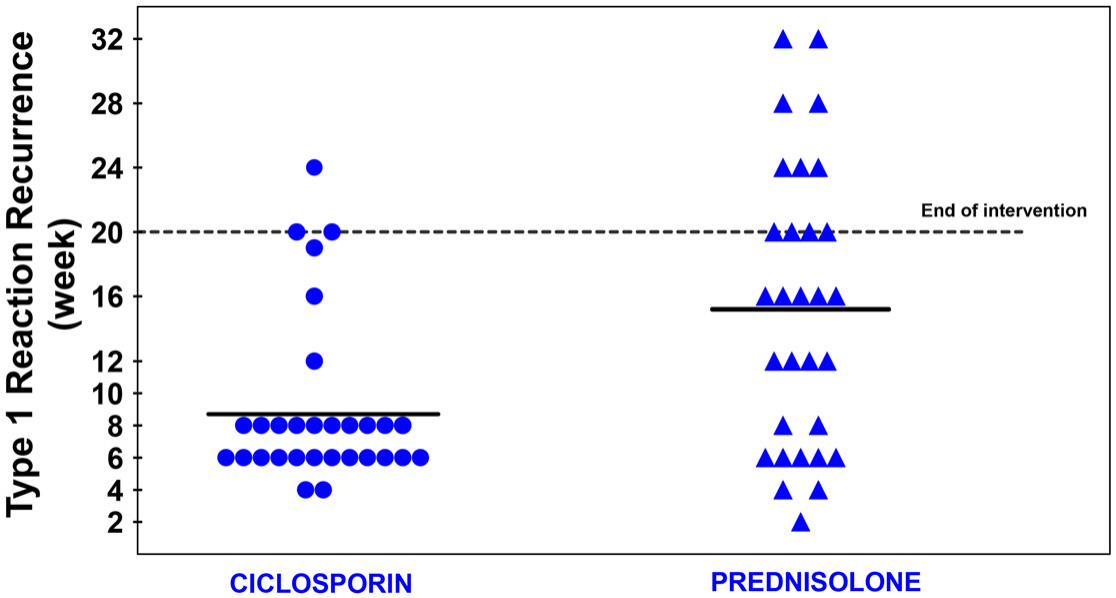
Time of first recurrence of T1R after initial control.

Fig 7 shows a cluster of T1R recurrence events around week 6 and week 8 in the ciclosporin arm patients. The analysis of Clinical Severity Score showed that at weeks 6 and 8, there was a statistical significant difference between the two treatment arms on the skin related A score. Prednisolone was given for the first four weeks of the study to patients on the ciclosporin arm to cover for the slow onset of action of ciclosporin. At week 4 prednisolone is stopped in these patients and many of them are having a flare-up of T1R at weeks 6 and 8, in particular in the skin signs of T1R, as shown on Fig 2.

#### Number of T1R recurrence episodes

The mean number of recurrence per patient was 1.35 (median 1) for the patients in the ciclosporin arm and 1.49 (median 1) for the patients in the prednisolone arm. There was no statistically significant difference between the two arms (Mann-Whitney U Test p = 0.365).

**Fig 8:**
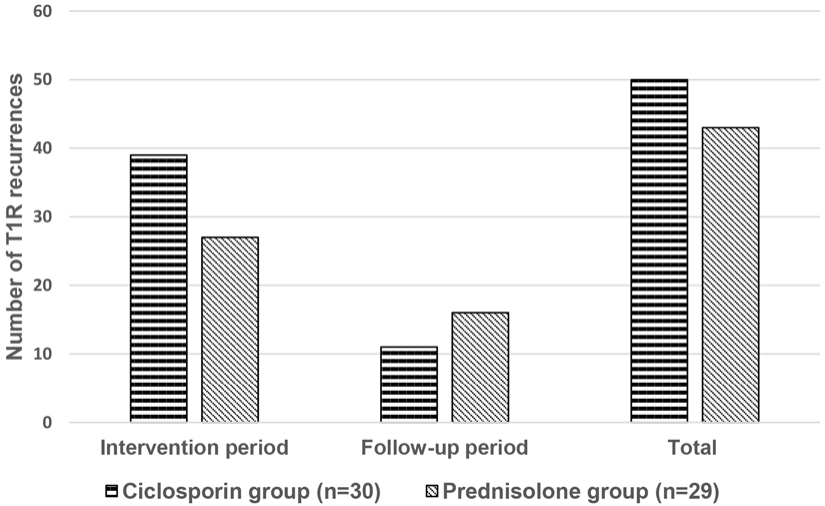
Number of T1R recurrence episodes per treatment arm.

A total of 93 episodes of T1R recurrence were experienced by the 59 patients. The largest difference in numbers of T1R recurrences occurs during the intervention period, with patients in the ciclosporin group experiencing 13 more recurrences than those in the prednisolone group (Fig 8). The difference in numbers of T1R recurrences within the intervention period or the follow-up period were not statistically significant.

#### Severity of T1R

**Fig 9:**
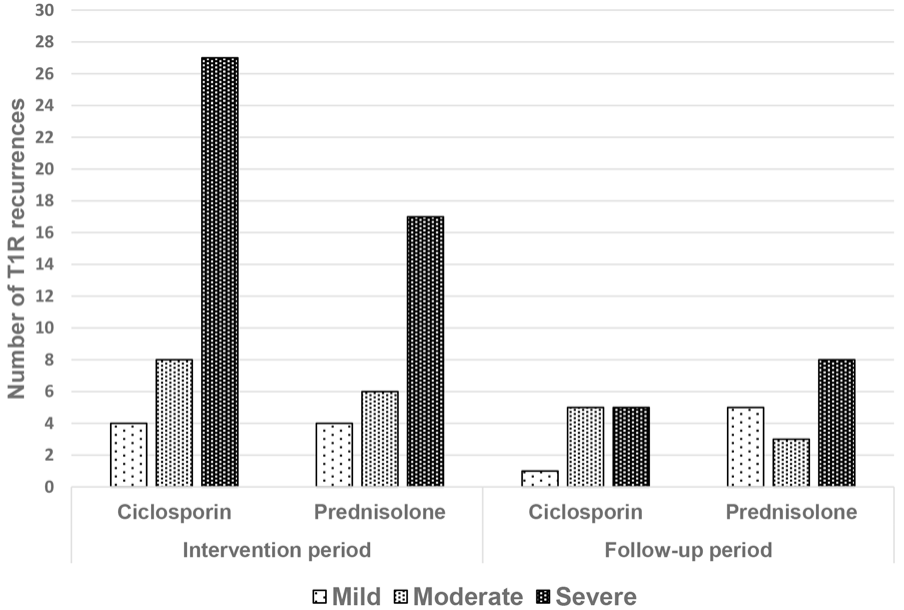
Number of T1R recurrence episodes by Clinical Severity Score.

The severity of the 93 episodes of T1R recurrence was graded using the Clinical Severity Score (Fig 9). In both intervention and follow-up periods, there was no statistically significant difference in the distribution of severity of recurrences between the two treatment arms, when graded by the Clinical Severity Score (Chi Square p = 0.926 and p = 0.162 respectively).

#### Amount of extra prednisolone

Table 6. shows the summary data for mean additional and total prednisolone received by all the patients recruited, per treatment arm.

**Table 6:** Additional and total prednisolone received in patients. (Group mean, range and median in mg.)

Period in study	Ciclosporin arm	Prednisolone arm	Whole group (n = 73)	P value (Mann Whitney U
	(n = 35)	(n = 38)		test)
**INTERVENTION**	**1608**	**559**	**1062**	**<0.000**
**PERIOD**	**(0–5705) 1400**	**(0–2030) 0**	**(0–5705) 840**	
**FOLLOW-UP PERIOD**	**1067**	**799**	**927**	**0.208**
	**(0–2870) 1260**	**(0–2310) 623**	**(0–2870) 980**	
**TOTAL STUDY PERIOD**	**2680**	**1358**	**1992**	**0.002**
	**(0–8085) 2520**	**(0–3710) 1435**	**(0–8085) 1820**	
**TOTAL PREDNISOLONE**	**3450**	**4208**	**3845**	**0.031**
	**(770–8855) 3290**	**(3010–6160) 4445**	**(560–8855)**	

Patients in the ciclosporin arm received significantly more additional prednisolone during the intervention period (p<0.000) and in the total study period (p = 0.002). Patients in the ciclosporin arm received 10% less steroid (mean 758mg) in total than the patients in the prednisolone arm.

Sixty patients in total received additional prednisolone during the study. Additional prednisolone was given for 91 T1R occurrences, as defined in the study protocol, and two for isolated nerve tenderness (Table 7). Twelve ENL episodes occurred in six patients during the study. Ten patients, five in each study arm did not require additional prednisolone.

**Table 7:** Reasons for additional prednisolone.

Reason for extra prednisolone	Ciclosporin arm	Prednisolone arm
**T1R (skin involved)**	**24**	**16**
**Neuritis/ NFI**	**27**	**26**
**ENL**	**4**	**8**

Excluding the patients with ENL does not alter the statistically significant differences seen in Table 6., in terms of additional prednisolone prescribed to each group.

Further sub-analysis showed that patients in the ciclosporin arm have more episodes of reaction recurrences requiring additional prednisolone. Severe recurrences involving skin flare-up are more frequent (16 vs 8) and occur more frequently in weeks 4 to 8 of the study.

**Fig 10:**
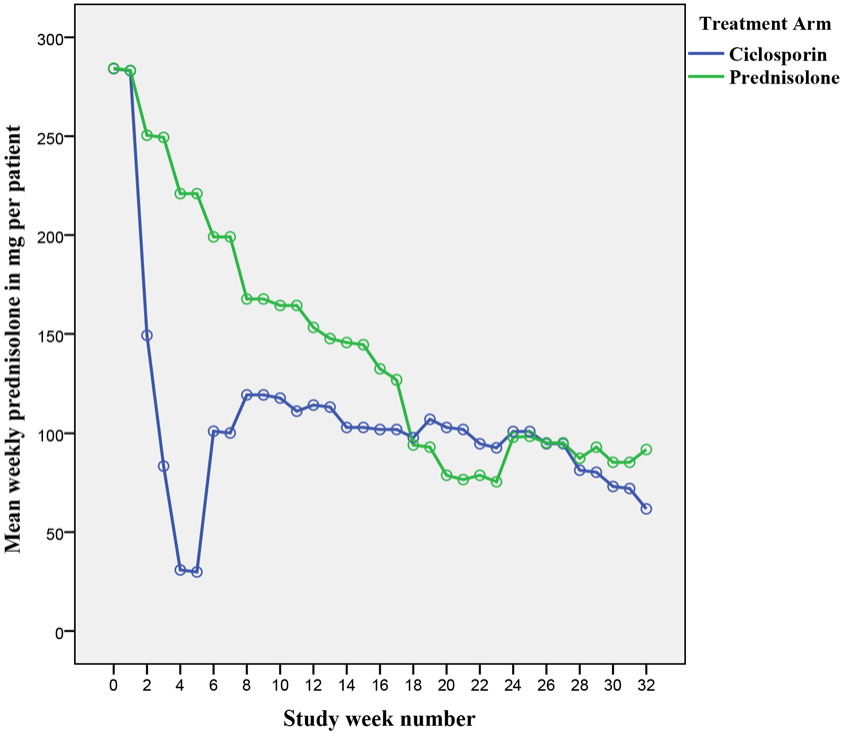
Weekly mean prednisolone per patient by treatment arm.

An ANOVA was conducted to get a clearer impression on the difference of mean prednisolone required by patients in both treatment arms throughout the different weeks in the study (Fig 10).

There was a significant difference (p = 0.003) in mean weekly prednisolone dose in both arms with time as less prednisolone was required by both groups as the study progressed. The week by week ANOVA breakdown shows the following important points:

- Weeks 4–15: significantly more prednisolone is taken by patients in the prednisolone arm
- Week 6: a sharp increase in prednisolone taken by patients on the ciclosporin arm is noted
- Week 20–24: significantly more prednisolone is taken by patients in the ciclosporin arm
- Week 24: an increase in the requirement of prednisolone is seen in patients on the prednisolone arm as flare-ups start to occur once the prednisolone regimen is stopped
- Week 29–32: at the end of the follow-up period, patients from the ciclosporin arm are on less prednisolone although this difference was not found to be statistically significant.

#### Adverse events

All the patients recruited to the trial experienced at least one adverse event during their period in the study. Patients experiencing minor and/or major adverse events that may be attributed to the study drugs are shown in Table 8. Patients are listed according to the study arm they were assigned to regardless of any additional prednisolone received during the study period to control any recurrence in reaction symptoms.

**Table 8:** Number of patients experiencing minor and major adverse events per study arm (related to ciclosporin and/ or prednisolone).

DRUG RELATED ADVERSE EVENTS		Ciclosporin arm (n = 35)	Prednisolone arm (n = 38)
**MINOR ADVERSE EVENTS**	**Moon Face**	**6**	**2**
	**Acne**	**10**	**13**
	**Fungal infections**	**10**	**8**
	**Gastric pain**	**19**	**14**
**MAJOR ADVERSE EVENTS**	**Infections**	**18**	**12**
	**Infected ulcers**	**14**	**14**
	**Hypertension**	**4**	**0**
	**Diabetes mellitus**	**1**	**1**
	**Nocturia**	**3**	**1**
	**GI bleeding**	**0**	**2**
	**Pulmonary tuberculosis**	**1**	**0**
**OTHER ADVERSE EVENTS**	**Headache**	**6**	**2**
	**Night sweats**	**3**	**3**
	**Hypertrichosis**	**1**	**0**
	**Gum hyperplasia**	**4**	**0**
	**Depression /anxiety**	**3**	**2**
	**Dysuria**	**2**	**0**
	**Vomiting**	**4**	**1**
	**Diarrhoea**	**3**	**5**
	**GI infection—bacterial**	**4**	**1**
	**GI infection—Giardia**	**3**	**4**
	**GI infection—H.pylori**	**2**	**4**
	**Blurred vision**	**2**	**3**
	**Conjunctivitis**	**3**	**3**

In this study, as patients in the ciclosporin arm receive 4 weeks of prednisolone at the start of the study, and further prednisolone for any flare-ups of reaction, it is misleading too associate the adverse event entirely with ciclosporin.

To address this and refine possible causative links, the adverse events association with either prednisolone or ciclosporin, were revised. Some adverse events are clearly related to one drug only, for example moon face and prednisolone, or gum hyperplasia and ciclosporin. Other adverse events can be caused by either of the two drugs. When the adverse event occurred after the end of ciclosporin treatment (week 21), it was attributed to prednisolone if the patient was on additional prednisolone. Adverse events occurring, in the ciclosporin arm, at a time when patients were receiving high doses of additional prednisolone were attributed to prednisolone. Any equivocal adverse events that can be related to both drugs were separated out. Table 9 suggests a higher rate of adverse events related to prednisolone than to ciclosporin.

**Table 9:** Number of adverse events attributable to ciclosporin and/or prednisolone.

DRUG RELATED ADVERSE EVENT		Ciclosporin related	Equivocal	Prednisolone related
**MINOR ADVERSE EVENTS**	**Moon Face**	**0**	**0**	**8**
	**Acne**	**2**	**8**	**13**
	**Fungal infections**	**1**	**5**	**12**
	**Gastric pain**	**3**	**6**	**24**
**MAJOR ADVERSE EVENTS**	**Infections**	**6**	**2**	**25**
	**Infected ulcers**	**3**	**10**	**40**
	**Hypertension**	**3**	**1**	**0**
	**Diabetes**	**0**	**1**	**1**
	**Nocturia**	**0**	**0**	**4**
	**GI bleeding**	**0**	**0**	**2**
	**Tuberculosis**	**0**	**1**	**0**
**OTHER ADVERSE EVENTS**	**Headache**	**5**	**0**	**3**
	**Night sweats**	**3**	**0**	**3**
	**Hypertrichosis**	**1**	**0**	**0**
	**Gum hyperplasia**	**4**	**0**	**0**
	**Depression /anxiety**	**1**	**1**	**3**
	**Dysuria**	**2**	**0**	**0**
	**Vomiting**	**1**	**0**	**4**
	**Diarrhoea**	**1**	**0**	**7**
	**Blurred vision**	**2**	**0**	**3**

There was no significant difference (p = 0.175) in the number of adverse events, classified by severity for each study arm (Table 10).

**Table 10:** Number of adverse events classified by severity.

		Ciclosporin (140)	Prednisolone (128)
**Severity of adverse event**	**Mild**	**58**	**66**
	**Moderate**	**70**	**49**
	**Severe**	**12**	**13**

Results of routine blood laboratory, excluding the patients who had a severe adverse event, were remarkably stable throughout the 32 weeks of the study. Seven patients had a drop in haemoglobin by at least 2 g/dL during their time in the study (three in the ciclosporin arm and 4 in the prednisolone arm). These patients had been started on MDT at the beginning of the trial and the haemoglobin drop was noted three months into the study. This is probably related to the dapsone in the MDT. Two patients had abnormal liver function tests at week 4 which resolved spontaneously. Renal functions (measured by serum creatinine and urea) and potassium levels were stable for all patients except in the four patients who experienced a serious adverse event (see below).

Six patients recruited with T1R went on to experience ENL during the study. Details of these patients are shown in Table 11.

**Table 11:** Patients in the T1R studies who experienced ENL.

**Patient number**	**Study arm**	**Week in study at ENL occurrence**	**R-J classification**	**BI at recruitment**
T1RA004	Cn	6	BL	2,3,2
T1RA041	P	6	BB	1,1,1
T1RA029	Cn	10	BL	2,3,4
T1RA036	P	16	BL	6,5,5
T1RA015	P	28	BL	5,5,6
T1RA053	P	28	BL	4,3,3

All these patients had a positive BI ranging between 1 and 6, and were categorised clinically as BB or BL. Patients experiencing both T1R and ENL can occur at this part of the spectrum which is known to be immunologically unstable.

Table 12 lists the five serious adverse events which occurred in this study. Three were definitely attributable to prednisolone and one definitely to ciclosporin. The fifth case, a patient diagnosed with pulmonary TB at week 22 (two weeks after stopping ciclosporin), may be attributable to both immune-suppressive drugs.

**Table 12:** Serious adverse events.

Age/ Sex	Study arm	Event wk no	Adverse event	Grad ing	Receiving pred	Pre-existing morbidity	Causality	Justification	Outcome
42/M	**Cn**	4	Severe headaches	3*	No	Severe headaches and visual blurring. Diagnosed with raised intra-cranial pressure.	Definitely related to ciclosporin	A rare but known side effect	Un-blinded. Ciclosporin stopped. Symptoms resolved. Continued on prednisolone
21/F	**Cn**	22	Pulmonary TB	4	Yes	Severe T1R necessitating high doses of additional prednisolone. Had 5705mg of additional prednisolone over 20 weeks	Definitely related to both drugs	Immuno-suppression caused by both ciclosporin and prednisolone	TB treatment given for 8 months No TB sequelae
58/M	**P**	2	Infective endophthalmitis	4*	Yes	Severe T1R –hospital admission, noted to have conjunctivitis and corneal ulcer. Right eye infection unresponsive to topical and oral treatment, progressed to endophthalmitis.	Most probably related to prednisolone	Immuno-suppression may have led to progression of infection	Un-blinded, right eye e-nucleation, withdrew from study, continued on prednisolone at Health Centre
54/M	**P**	24	Death	5	Yes	Severe T1R, osteomyelitis, septicaemia and anaemia- all treated week 22. On additional prednisolone (2015mg over 24 weeks, total 5025mg) and proton pump inhibitor for severe dyspepsia.	Definitely related to prednisolone	Developed acute abdomen after severe dyspepsia. Possible perforated gastric ulcer and multi-organ failure	Death
24/M	**P**	26	Facial cellulitis	3	Yes	Dental abscess–progressed to facial cellulitis	Most probably related to prednisolone	Immuno-suppression	Recovered

Cn: ciclosporin arm; P: prednisolone arm

* Un-blinded

Grading: 1 = Mild; 2 = Moderate, 3 = Severe; 4 = Life-threatening or disabling; 5 = Death (according to National Cancer Institute adverse event grading system–CTCAE)

#### Quality of life

Patients completed our validated SF-36 health related quality of life questionnaire in Amharic at recruitment and at the end of the study. Of the initial 35 patients in the ciclosporin arm and the 38 in the prednisolone arm, 31 and 27 respectively completed the end of study questionnaire. No significant difference was detected between the changes in score for each study arm.

**Fig 11:**
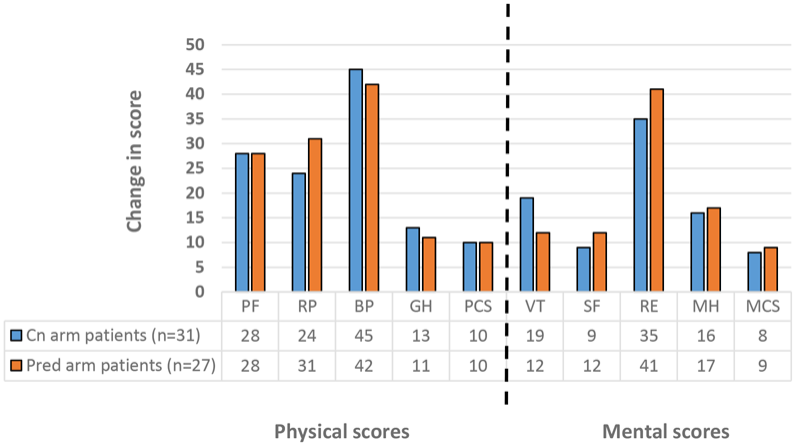
Change in SF-36 scores between start and end of T1R study. PF-physical functioning, RP-role physical, BP-bodily pain, GH-general health perceptions, VT-vitality, SF-social functioning, RE-role emotional, MH-mental health, PCS-physical component summary, MCS-mental component summary.

Table 13 shows the mean group score for each SF-36 scale at the start and at the end of the study, divided by treatment arm. The difference in group score between baseline and end of study is shown as the effect and the size of this effect is calculated and described. Standardised mean differences of less than 0.30 standard deviations are small effects, 0.30–0.80 are moderate, and more than 0.80 are large. All the scores were significantly increased (p<0.05) between the start and the end of the study except for the social functioning scale (SF) in both treatment arms. The changes in score in each scale, mostly with moderate and large effect size, are shown graphically in Fig 11. The largest score increase was in the bodily pain scale and the emotional role.

**Table 13:** Mean group SF-36 scores and the effect in score difference.

**Patients on Ciclosporin Arm**						
SF-36 variables	Baseline Mean ± SD	End of study Mean ± SD	Effect (Difference = end of study—baseline)			*p value* (paired sample t test)
			Mean ± SD	ES	ES description	
PF	50.8 ± 32.1	78.9 ± 20.3	28.1 ± 34.2	0.82	large	.000
RP	31.9 ± 27.2	55.8 ± 27.2	24 ± 36.3	0.66	moderate	.001
BP	20.5 ± 15.4	65.5 ± 30.7	45.1 ± 33.6	1.34	large	.000
GH	32.1 ± 18.8	45.3 ± 19.6	13.2 ± 23.4	0.56	moderate	.004
VT	38.1 ± 17.7	56.7 ± 20.4	18.5 ± 21.2	0.88	large	.000
SF	71.0 ± 37.0	80.2 ± 29.0	9.3 ± 38.6	0.24	small	.191
RE	28.0 ± 29.3	62.6 ± 32.8	34.7 ± 36.1	0.96	large	.000
MH	41.1 ± 22.7	57.4 ± 22.1	16.3 ± 27.1	0.6	moderate	.002
PCS	36.9 ± 7.2	47.4 ± 6.7	10.5 ± 9.8	1.06	large	.000
MCS	35.1 ± 10.3	43.2 ± 11.5	8.1 ± 11.9	0.68	moderate	.001
**Patients on Prednisolone Arm**						
SF-36 variables	Baseline Mean ± SD	End of study Mean ± SD	Effect (Difference = end of study—baseline)			*p value* (paired sample t test)
			Mean ± SD	ES	ES description	
PF	54.3 ± 35.7	82.0 ± 20.1	27.8 ± 43.0	0.65	moderate	.002
RP	34.3 ± 31.6	64.8 ± 20.5	30.6 ± 38.7	0.79	moderate	.000
BP	28.9 ± 23.4	70.4 ± 25.6	41.5 ± 34.0	1.22	large	.000
GH	39.8 ± 18.6	50.3 ± 20.0	10.6 ± 21.2	0.5	moderate	.015
VT	48.8 ± 19.9	60.6 ± 19.1	11.8 ± 25.3	0.47	moderate	.023
SF	74.1 ± 33.2	85.6 ± 26.6	11.6 ± 41.6	0.28	small	.160
RE	33.3 ± 29.9	74.7 ± 22.2	41.4 ± 39.9	1.04	large	.000
MH	45.9 ± 21.7	63.3 ± 14.9	17.4 ± 22.8	0.76	moderate	.001
PCS	38.9 ± 9.8	48.6 ± 7.0	9.7 ± 12.5	0.78	moderate	.000
MCS	38.0 ± 10.4	47.0 ± 6.7	9.0 ± 10.2	0.88	large	.000

*PF-physical functioning*, *RP-role physical*, *BP-bodily pain*, *GH-general health perceptions*, *VT-vitality*, *SF-social functioning*, *RE-role emotional*, *MH-mental health*, *PCS-physical component summary*, *MCS-mental component summary*

SD = standard deviation

ES = effect size = mean (effect)/ SD (baseline)

## Discussion

This is a large RCT done on the treatment of leprosy T1R in Africa and the first to compare the efficacy of ciclosporin to prednisolone. The participants were closely monitored using established tools to measure outcomes. This is the first time that leprosy patient participant quality of life has been assessed in a trial. We shall discuss aspects of the study design that may have made the study more complex

We conducted a non-inferiority trial because we anticipated finding that ciclosporin and prednisolone was not worse than prednisolone alone, in the treatment of T1R. We hoped that the steroid sparing effect of ciclosporin may be strong enough to decrease the rate of steroid-related side effect. A sample size of 48 patients per treatment arm was calculated with the assumption that prednisolone, based on previous studies identified in the literature review, leads to an improvement of about 60% in nerve function in patients with new T1R. The non-inferiority margin of 0.25% was selected. Our study recruited a total of 73 patients, 35 in the ciclosporin arm and 38 in the prednisolone arm. This smaller sample size reduces the power to detect a significant difference in the study from 80% to 70%.

At the end of the 20-week intervention, both groups of patients recruited to either the ciclosporin and prednisolone arm or the prednisolone only arm had similar improvement rates in T1R severity as assessed by the Clinical Severity Score. Both groups of patients also showed an improvement in clinical outcomes as assessed by the physician. In patients receiving ciclosporin and prednisolone 100% of skin lesions recovered or improved, 75% of motor nerves improved or recovered, and 66% of sensory nerves improved or recovered. In comparison, for patients receiving prednisolone only, we found that 94% of skin lesions had recovered, 74% of motor nerves had improved or recovered, and 49% of sensory nerves had improved or recovered.

Skin lesions in patients on the ciclosporin and prednisolone arm flared up around weeks 6 and 8 of the study, just after the prednisolone cover was stopped at week 4. This suggests that the therapeutic level for ciclosporin had not been reached when the prednisolone was stopped. In the study design it was assumed that four weeks of initial prednisolone would adequately cover the slow onset of action of ciclosporin. Several problems can be identified in retrospect with this regimen. The onset of action of ciclosporin is reported to be between four to eight weeks [21], so potentially stopping the adjunctive prednisolone at week 4 was too early. Continuing prednisolone cover a bit longer may have prevented these early flare-ups in patients on ciclosporin.

Patients in the ciclosporin arm had fewer flare-ups in skin reaction signs during the follow-up period. This may be because that the dose of prednisolone in these patients was higher in the early follow-up period as a result of additional prednisolone given for earlier flare-ups and therefore providing an extended protective effect. Patients in the prednisolone only group tended to have flare-ups in skin lesions towards the end of the intervention period and in the follow-up period as the dose of prednisolone was decreased or stopped.

In patients in both study arms, nerves reported to have been impaired for less than 6 months showed a good improvement rate in motor function (Cn 74% and P 68%) and in sensory function (Cn 70% and P 56%). Patients who received ciclosporin and prednisolone had better improvement in nerve function impairment than those who received prednisolone only. Improvement in nerve function in patients on the prednisolone only arm are similar to those reported in previous studies. In an open Bangladeshi study (n = 132), it was reported that 68% of sensory nerves and 67% of motor nerves improved after a 16-week course of prednisolone [8]. In the methylprednisolone trial, 70% of the patients with T1R (n = 42) who completed a 16 weeks course of prednisolone showed improved nerve function [16].

As in previous studies, an important 24 to 32% of nerves did not improve with treatment. It may be that a proportion of these nerves with no improvement had been affected for longer than six months or that the poor response to treatment may be due to physiological factors.

Between 36% and 46% of nerves that patients reported as having been affected for longer than six months improved in both sensory and motor function. Evidence for the six-month cut-off often used in deciding whether nerve function impairment should be treated with steroids is based on one TRIPOD study only [22]. Patients’ accuracy of recall with regards to the length of time the NFI has been present is problematic and may cause bias. Most of the patients in this T1R study had not been diagnosed or not been previously seen at ALERT clinic, so that no previous VMT/ST assessments were available for comparison and dating of NFI. It is also difficult for patients to be exact about timing of sensory and motor NFI, especially when subtle changes can go unnoticed.

The timing of the first episode of T1R recurrence was significantly earlier for T1R patients on ciclosporin and prednisolone (median 8 weeks) than those on the prednisolone only (median 16 weeks). This reflects the earlier mentioned increase in skin reaction a week or two after the prednisolone cover is stopped in the patients on ciclosporin. The mean and median number of recurrences per patient was not significantly different between patients on the two study arms. More T1R recurrence episodes occurred in the intervention period in the patients on ciclosporin but the severity of these recurrence was not significantly different from patients on prednisolone only. Ten patients, five in each arm of the study, had no T1R recurrence throughout the 32 weeks in the study.

In our study, 85% of patients had a T1R recurrence. The proportion of patients with T1R recurrence was similar for both study arms. This is a very high recurrence rate. In the Marlowe study, patients treated with ciclosporin had a recurrence rate of 50% in skin lesions, 71% in sensory nerve impairment and 67% in motor impairment [13]. In TRIPOD 2 [23], 27% of patients with mild sensory impairment treated with prednisolone experienced deterioration necessitating additional prednisolone. In the Methylprednisolone study, 45% of patients on methylprednisolone and 50% of patients on prednisolone only required additional prednisolone for either skin or nerve deterioration [16]. In the Indian RCT looking at three different prednisolone regimens, the proportions of individuals with T1R or NFI of less than three months duration requiring additional prednisolone in the three groups was 24%, 31%, and 46% respectively. Individuals who received prednisolone for five months were significantly less likely to require additional prednisolone [24]. It is difficult to know whether the higher rate of recurrences in our study may be due to difference between Ethiopian and Indian or Nepalese patients, but the Marlowe study on ciclosporin which compared two groups did find that Ethiopians patients had a higher rate of T1R relapse compared to Nepalese patients [13].

Significantly more additional prednisolone was required by patients in the ciclosporin arm both during the intervention period and the full 32 weeks of the study. Mean total weekly prednisolone received by patients on the ciclosporin arm was lower than that received by patients on the prednisolone arm throughout the study except for the period week 18 to 25. In total, the ciclosporin group received 10% less total prednisolone (p = 0.031). The magnitude of this steroid sparing effect does not seem important enough to give a patient with T1R a 20-week course of an additional immune-suppressive drug such as ciclosporin unless a large difference in improvement of nerve function or in the rate adverse events is noted between the two treatment groups.

More minor, major and serious adverse events were directly attributable to prednisolone than to ciclosporin. Our adverse effects rates attributable to ciclosporin was similar to that in the previous ciclosporin only trials in T1R [13] and to those reported by the drug manufacturers. Six patients recruited with T1R went on to experience ENL during the study. Although it is known that patients in the BB-BL-LL spectrum of leprosy may develop both T1R and ENL simultaneously and/or alternatively, there is little published data on the frequency and risk factors for this phenomenon. Our data suggests that this is commoner than recognised.

This is the first time that the SF-36 Health Related Quality of Life questionnaire has been used in a leprosy clinical trial. All the comparisons were done on group mean quality of life scores and not on individual patient scores. There was no statistically significant difference in changes in all scores between patients on the ciclosporin arm and those on the prednisolone arm.

All the scores were significantly increased (p<0.05) between the start of the study and the end of the study except for the social functioning scale (SF). This means that both groups of patients improved significantly after treatment with both treatments. Statistically significant differences, however, do not imply that a meaningful or relevant difference has been demonstrated for the individuals enrolled in such trials[25].

To determine whether the observed changes in SF-36 scores were statistically and clinically meaningful, minimal clinically important changes (MCIC) for SF-36 subscales are needed. MCIC have not been studied in leprosy reactions so the closest we can come to defining these is by using the published standards for minimal "clinically and socially relevant" change in group scores as a measure of MCIC at a group level [26]. Using these criteria, all the scores of the SF-36 scales improved by at least 5 point in the patients randomized to both treatment arms, indicating that the improvement in quality of life was clinically and socially relevant, for both groups with no significant difference between the two groups.

### Conclusions

This study is the first double-blind RCT assessing ciclosporin, a potent immunosuppressant in the management of T1R. All the patients with T1R treated with ciclosporin and prednisolone or with prednisolone alone improved in all three Clinical Severity Score components. There was no statistically significant differences between the two study arms, suggesting that treatment of T1R with ciclosporin and prednisolone in non-inferior to prednisolone alone.

Recurrences of T1R were equally frequent in both treatment arms. These recurrences were treated with additional prednisolone. The patients on the ciclosporin arm of the study received 10% less steroids than those on the prednisolone only arm during the 32 weeks of study.

This study has shown that the steroid-sparing effect of ciclosporin is limited. The pilot study done by Marlowe in 2007 suggested that ciclosporin may be as efficient as prednisolone in the treatment of T1R. The study designs are different and no additional prednisolone was given in the Marlowe study for T1R or NFI flare-up; the dose of ciclosporin was increased in such cases.

In view of the fewer side effects of ciclosporin compared to prednisolone, ciclosporin could be a useful safe alternative second-line drug for patients with T1R in whom prednisolone is not effective, or is causing adverse events. We would recommend that ciclosporin be prescribed in conjunction with oral prednisolone (unless the latter is contraindicated) for the initial eight weeks. Presently a 20 week course of ciclosporin for a patient in the weight range of 40–49kg, would cost USD 820, compared to a course of prednisolone costing USD 10.

This study has highlighted that corticosteroid treatment for T1R and NFI is sub-optimal even when given in a standard reducing course over 20 weeks. The TENLEP multi-centre RCTs are comparing a 32-week vs 20-week course of prednisolone for NFI [18]. This would mean a cumulative dose of prednisolone greater than 5grams compared to 3.5grams over 20 weeks recommended by Rao [24]. The development of more prolonged treatment protocols would require careful monitoring of adverse events and in particular the long term sequelae of corticosteroid therapy.

This study illustrates the difficulty in switching off leprosy inflammation. Better treatment agents for reactions and nerve damage are needed. Clinical studies in T1R should be accompanied by laboratory based research to investigate the mechanisms of inflammation in T1R, identify patients at risk of recurrences and possibly identify a better agent for the treatment of T1R.

## Supporting Information

S1 File CONSORT checklist. (DOC)
